# The Medical Association Banquet

**Published:** 1887-05

**Authors:** 


### Editorial.

### THE MEDICAL ASSOCIATION BANQUET.

The banquet given the Association by the physicians of Atlanta was a most enjoyable occasion, and formed a fitting climax to the series of hospitalities that Atlanta proffered to the visiting doctors. The toasts were so fittingly chosen, and answered so well, that comparison would be invidious. They were all delightful, witty, humorous and entertaining. Among so many it is impossible to single out one particularly bright, but the response to "The Press," by Mr. F. H. Richardson, the well-kown Washington correspondent of the Atlanta *Constitution*, sounded so clear a note on a needed subject that it is given entire. The remarks apply not only to the press in general, but would be of benefit to the medical press. The standard of veracity and honesty in medical journals could well be raised without serious danger and to the benefit of the profession.

"The Press: Aggressive, enterprising, sensational, indispensable; may its veracity never grow less."

Concerning the terms of the first proposition of your toast, there can be no disputation, at least on this occasion. If I do not naturally possess it, I can borrow from the traditions of my guild the modesty to admit that the press is "aggressive, enterprising, sensational, indispensable."

The only possible issue must, therefore, be joined on your last proposition, in which the velvet of a benevolent invocation hardly sheathes the claw of a somewhat tigerish innuendo.

"The veracity of the press!" If I were to proceed according to the approved fashion of the schools, I should first attempt an analysis of veracity in general. This would involve a consideration of the subject in its widest scope. It would include even the veracity of the medical profession. Mr. President, do doctors always tell the truth ? But you are not on trial here. Wrapping yourselves in that mantle of Pharisaical complacency which every profession is too prone to wear, you tacitly assume a virtue which you deny to the poor publican, whom you have this evening admitted within your hospitable gates.

Before you condemn the press, I beg you to remember that veracity, in its essence, is a rare commodity in this sin-stricken world. Too often have Truth and Falsehood met and kissed each other. In our affairs these strains are so often intermingled, and the pure blood of either line is so hard to find, that Thomas Carlyle, as loyal a lover of the truth as ever lived, actually became enamored of the personality of the most audacious of falsifiers. In his sketch of Count Cagliostro he says· "If we cannot have a truth-speaker and truth-doer, let us at least have the melancholy pleasure of beholding a decided liar."

I cannot claim for the press the benefit of this unique eulogium. The fact is that the press can tell the truth or its opposite, just as it pleases; and that man has not yet been born who can prognosticate on a given occasion which it will choose.

Charles Lamb said that Wordsworth could write as good a play as "Hamlet" "if he had a mind to." The press could be as veracious as a doctor "if it had a mind to."

But, seriously, gentlemen, you must remember what sort of world the press has to deal with. Never was lover compelled to be more assiduous to his mistress than is the press to the public. The public can demand and can get just such newspapers as it wants, and it lavishes its favors on some very bad ones.

I can imagine a higher incentive to veracity in the press than the fact that the English journal which, for several generations past, has been most reliable, has been eclipsed in circulation, if not in influence, by at least a dozen of its less scrupulous contemporaries, while the New York newspaper, which stands first for veracity, ranks as low as tenth in circulation. If veracity were more popular, the press would he more veracious. I admit that this does not excuse the press. If the public insists on being wicked, the press cannot, on that account, find a justification for being wicked also, and it is but fair to admit that just censure lies at the door of the press in this matter. Some of our most entertaining newspapers are the least reliable; some of our purest newspapers are dismally dull. Some time people have a way of making the truth so unattractive that I do not wonder at the popularity of well-spiced falsehood. There is no reason why the journal of the highest tone and the most incorruptible principles should not also be the brightest and most readable. We must divorce truth from stupidity and marry her to enterprise. But there are many things about the press besides its devotion to veracity which are commendable. The day of great editors, of gigantic and absorbing personalities, has passed. The press is no longer a pretentious oracle. It has become a practical factor in government, in politics, in thought in every direction. It permeates society and peeps in at all its proceedings. It tracks the lonely waste; it blazes the wild with the pioneer; it penetrates the jungle with the explorer; it cuts the ice of the arctic; it ploughs the burning sands beneath the line; the statesman cannot escape its vigils; the tramp is the subject of its care; it stands beside the physician in pestilence; it sits with the millionaire in his palace; it swelters with the wretch in his hovel. Wherever men strive, and suffer, and die, the man of the press is, or ought to be. Such tests develop in him the frailties which are common to humanity; they bring out in him the strength and splendor which give to men their noblest virtues. Not always among the names highest on the roll of journalistic fame are these virtues most illustriously developed. They were lowly men of whom it was said that the world was not worthy.

I mind me of some instances of unheralded heroism which make me proud that I belong to the same clan that gave their examples to the world.

I remember poor Donovan, who went for a London newspaper into the Soudan that he might tell luxurious England what her army was doing in that wild land. Lost, broken in strength, yet brave in spirit, he finally found himself beside one of those beautiful lakes, which nature sometimes sets as a sparkling mockery amid her most desolate wastes. Lying there without hope of rescue, weak and starving, as he awaited the inevitable end, he wrote his last lines. They were no lament for his fate, no wail of woe. They were words of cheery friendship to an old companion away off in happy England, more mindful of his friend's fancies than of his own pangs. "Dear Jack," he wrote, "how I wish I could catch and send you one of these little red monkeys which come down to the lake to drink, and one of these bright-plumaged birds which are flitting about me—" The sharp finger of death made the period.

I think of that New York boy who, two years ago, crossed the ocean that he might reach Marseilles when people were flying from it by the thousand because cholera had set its black pall over the city. And how, day after day, he cabled the best and truest accounts of the progress and nature of the disease; how without fear he looked death straight in the eyes until he fell beneath the scourge and sent his last dispatch on the day he died.

I cherish, with no feeling short of reverence, the memory of my friend, Herbert Landrum, who in 1878, when Memphis was smitten of yellow fever, said good-bye to all his loved ones and never once thought of leaving his post of duty. I remember how, during all the long hours of the hot and direful days, he battled side by side with men of your profession against the pestilence, devoting the hours that should have been for rest to service in hospitals, and how, away into the still, awful nights, he sat toiling at his desk to tell the world the dreadful story, until at last his kind ministering hands grew hot and heavy, his stainless pen faltered and stopped on its last page, and his great, golden heart was hushed forever.

Such are the men who make every calling high. Such are the men who ennoble every profession. Such are the men who keep the earth sweet.

The press is not all it should be. Which of our professions is beyond improvement in any respect? I trust the press may rise to a truer conception of its duty, to a more faithful performance of it. I hope that you and I may live to see the day when its code of morals shall be as pure and its plane of action as high as the counsel which William Makepeace Thackeray gave to the press club of London when he exclaimed:

"Ye knights of the pen! ye knights of the pen! Let honor be your shield and truth tip your lances. Be gentle with women. Be tender to children. Be true to all things that are true, and vow yourselves to eternal warfare against corruption and wrong."

---

